# Clinical and genetic analysis of patients with Sjögren-Larsson syndrome in China

**DOI:** 10.1186/s13023-026-04489-x

**Published:** 2026-07-16

**Authors:** Feng Chen, Bowen Yu, Yangshuo Wang, Liu Yuan, Liwei Zhang, Tinghong Liu, Feng Zhai, Jinshan Xu, Xin Ni, Shuli Liang

**Affiliations:** 1 Department of Functional Neurosurgery, Beijing Children’s Hospital, Capital Medical University, National Cener for Children’s Health, Beijing, China; 2 Key Laboratory of Major Diseases in Children, Ministry of Education, Beijing, China; 3 Department of Otolaryngology, Head and Neck Surgery, Beijing Children’s Hospital, Capital Medical University, National Center for Children’s Health institution, Beijing, China

**Keywords:** Sjögren-Larsson syndrome, *ALDH3A2* gene, Ichthyosis, Intellectual disability, Spastic diplegia

## Abstract

**Objective:**

To characterize the clinical features and genetic spectrum of Chinese patients with Sjögren–Larsson syndrome (SLS).

**Methods:**

We retrospectively reviewed genetically confirmed SLS cases managed in the Functional Neurosurgery Department of Beijing Children’s Hospital. We also searched Chinese- and English-language databases to identify additional SLS cases for comparative analysis. Structural effects of detected variants on fatty aldehyde dehydrogenase (FALDH) were explored using PDB-derived models and PyMOL.

**Results:**

Three SLS patients were identified, including one carrying a novel variant not previously reported. Literature review yielded 19 additional Chinese cases. Globally, SLS has a broad distribution, with the highest case counts reported in the United States, Sweden, and China. Compared with cohorts from other countries, Chinese patients showed a higher proportion of females, and compound heterozygous variants were more frequent than homozygous variants. The c.1157A > G substitution emerged as the most common variant in Chinese SLS. Three-dimensional modeling suggested that several variants likely perturb FALDH conformation.

**Conclusion:**

Chinese SLS patients exhibit distinctive clinical and genetic patterns relative to other populations, with c.1157A > G being the most frequent variant. Structural modeling supports a potential conformational impact of these variants on FALDH.

**Supplementary information:**

The online version contains supplementary material available at https://doi.org/10.1186/s13023-026-04489-x.

## Introduction

Sjögren–Larsson syndrome (SLS) was first described by Sjögren and Larsson in 1957 and is classically characterized by the triad of ichthyosis, intellectual disability, and spastic diplegia or quadriplegia [1]. Additional, frequently observed features include ocular abnormalities (reduced vision, photophobia, crystalline macular/retinal degeneration), prematurity, epilepsy, and white-matter abnormalities [2]. SLS is now recognized as an autosomal recessive hereditary neurocutaneous disorder, caused by mutations in the *ALDH3A2* gene located at 17p11.2 [3]. Over a hundred pathogenic mutations have been identified that can cause SLS, including missense mutations, frameshift mutations, splice site mutations, deletion-insertion mutations, deletion mutations, nonsense mutations, and large deletion rearrangements [4]. The *ALDH3A2* gene encodes fatty aldehyde dehydrogenase (FALDH), which is part of nicotinamide-adenine dinucleotide oxidoreductase (FAO) enzyme complex. FAO comprises two enzymatic activities-fatty alcohol dehydrogenase and FALDH-that sequentially oxidize fatty alcohols to fatty aldehydes and then to fatty acids. FALDH, as part of FAO, mainly functions to catalyze the oxidation of fatty aldehydes into fatty acids. FALDH deficiency disrupts lipid metabolism, which is thought to underlie SLS pathophysiology. Fatty alcohol accumulation in the skin disrupts the epidermal water barrier, causing moisture loss from the epidermis and resulting in ichthyosis [5]. Abnormal lipid metabolism and fatty alcohol accumulation can damage the myelin structure of the central nervous system, leading to white matter abnormalities and neurological disorders [6]. There is currently no curative therapy; most individuals survive long-term but experience substantial morbidity and reduced quality of life. Although SLS is relatively rare, it is widely distributed across the world. The global variation in the number of reported cases may be related to multiple factors, such as the level of medical care, population size, and ethnic distribution.

In this study, we retrospectively identified three patients with SLS, including one with a previously unreported variant. Through database searches, we mapped the global distribution of reported SLS cases and found that c.1157A > G (p. Asn386Ser) is the most common variant in China. We further modeled the three-dimensional structure of FALDH harboring this variant to explore its potential pathogenic mechanism, providing a basis for future mechanistic studies and potential therapeutic targeting.

## Methods

We reviewed three patients with Sjögren–Larsson syndrome (SLS) admitted to the Functional Neurosurgery Department of Beijing Children’s Hospital between 2024 and 2025. Detailed clinical data were extracted from medical records, including sex, medical and perinatal history, family history, physical and neurological examination findings, electroencephalography, brain magnetic resonance imaging, medications, and treatments. Follow-up was conducted via outpatient visits or telephone calls.

For genetic testing, 3–4 mL of EDTA-anticoagulated peripheral blood was collected from each proband and their parents. Genomic DNA was extracted and subjected to whole-exome sequencing (WES). Candidate variants were amplified by polymerase chain reaction and confirmed by Sanger sequencing. Variants were annotated to the GRCh37/hg19 reference genome using ALDH3A2 transcript NM_000382.3. The pathogenicity of variants was assessed according to the 2015 guidelines from the American College of Medical Genetics and Genomics [7]. The gene sequencing was entrusted to Beijing MyGenostics, Co., Ltd., and Cipher Gene. Data analysis was performed by Key Laboratory of Major Diseases in Children, Ministry of Education, Beijing.

To comprehensively identify SLS cases in China, we systematically searched two English-language and three Chinese databases: Leiden Open Variation Database (LOVD) [8], PubMed, China National Knowledge Infrastructure (CNKI)，the Wanfang Data Knowledge Service Platform, and VIP Chinese Journal Service Platform. Cases from other countries were retrieved from LOVD.

For structural analysis, published three-dimensional structures of FALDH from the RCSB Protein Data Bank [9] were visualized, and local molecular interactions around variant residues were modeled using PyMOL v3.3.6.1.

## Results

### Clinical features of three patients

P20, a male, was born prematurely at 36 weeks of gestation. Approximately one month after birth, he developed ichthyosis-like skin changes, primarily affecting the limbs and trunk. By one year of age, motor development was markedly delayed, prompting initiation of rehabilitation therapy. There was no history of epilepsy. At 4 years and 11 months, evaluation at our hospital revealed moderate intellectual disability; he was able to walk independently but displayed a spastic gait, with increased muscle tone in all limbs, more pronounced in the lower extremities. Cranial MRI was unremarkable, while electroencephalography showed intermittent focal discharges. Both parents had normal phenotypes. Genetic testing confirmed SLS, identifying a homozygous c.1157A > G variant inherited from each parent.

P21, a female, was born prematurely at 31 weeks of gestation. Soon after birth, she exhibited dry skin, and by 2 months developed ichthyosis-like changes involving the limbs and trunk. Motor development was significantly delayed by 1 year of age, and rehabilitation therapy was initiated. She had a history of two febrile seizures. At 2 years and 10 months, evaluation showed severe intellectual disability; she was unable to stand or walk independently. Muscle tone was normal in the upper limbs but markedly increased in the lower limbs. X-ray imaging demonstrated bilateral developmental hip dysplasia. Fundus examination revealed bilateral macular degeneration. Cranial MRI demonstrated symmetrical patchy signal abnormalities in the frontal and parietal lobes and around the third ventricle, appearing hypointense on T1-weighted imaging and hyperintense on T2-weighted imaging. Both parents had normal phenotypes. Genetic testing confirmed SLS, identifying compound heterozygous variants—c.779delA inherited from the father and c.1157A > G from the mother.

P22, a male, was born prematurely at 36 weeks of gestation. Around 1 month after birth, he developed ichthyosis-like skin changes involving the limbs and trunk. At 10 months, his parents observed significant motor delays and increased muscle tone in the lower limbs. He was treated with botulinum toxin injections and rehabilitation therapy, with mild improvement. There was no history of epilepsy. At 2 years and 10 months, examination revealed intellectual disability; he could walk independently but had an unsteady gait. Muscle tone was normal in the upper limbs but markedly increased in the lower limbs. Fundoscopic examination, electroencephalography, and cranial MRI showed no remarkable abnormalities. Genetic testing confirmed SLS due to a homozygous c.313_327delinsTG variant, which has not been previously reported. Both parents carried the heterozygous variant at the same locus.

### Comparison of SLS patients in China and other countries

**Fig. 1:**
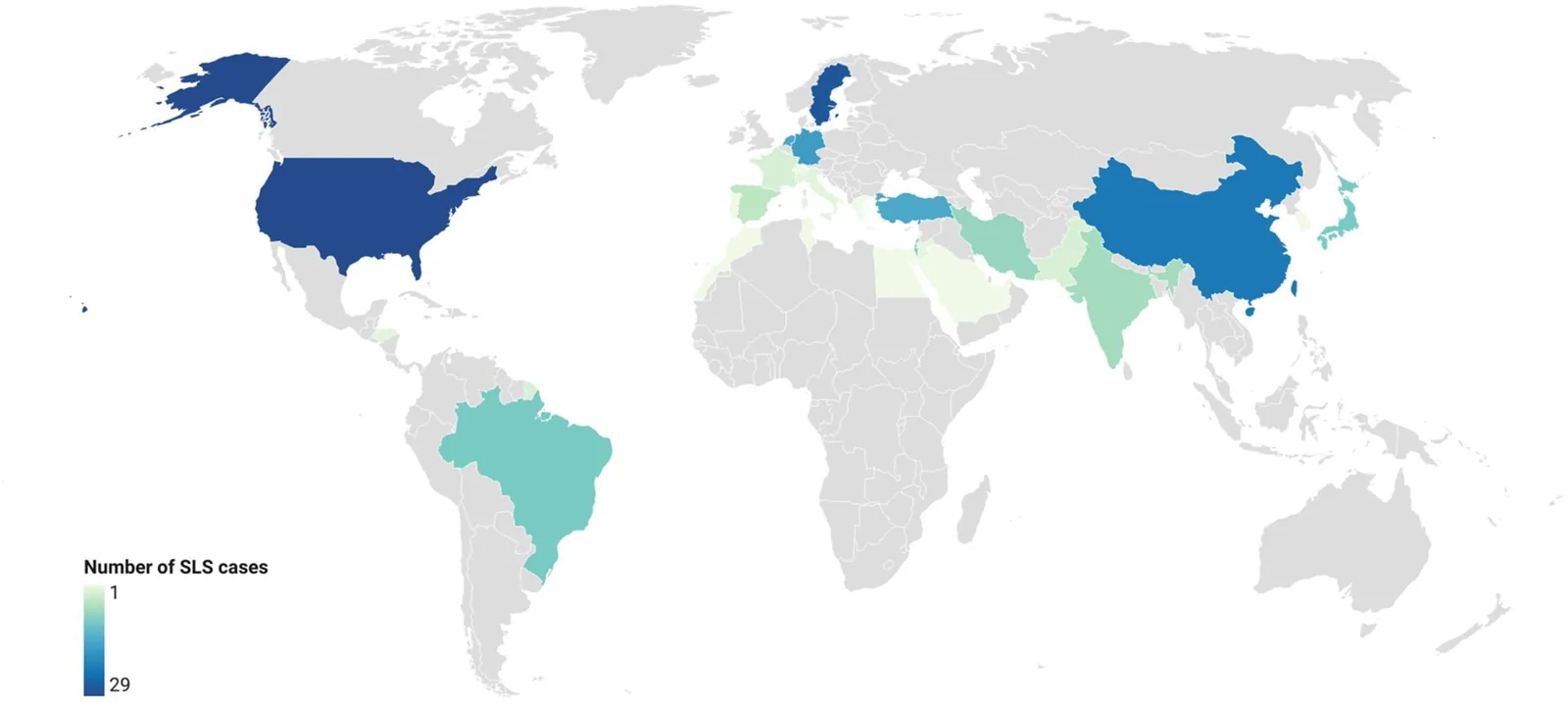
Geographic distribution of 200 patients with Sjogren-Larsson syndrome

By searching relevant databases, we identified 22 Chinese patients with SLS [10–21], including the 3 cases reported in this study (Table 1), and 178 SLS patients from other countries (Supplement Table) were included. Cases lacking nationality information or complete genetic data were excluded. The global distribution of all 200 patients is shown in Fig. 1. The United States, Sweden, and China have the highest numbers of reported cases.

**Table 1:** Clinical and genetic features in 22 Chinese patients with SLS

Patient	Gender	Age at the time of genetic testing	Mutation at cDNA level	Mutation at protein level	Zygosity	Ichthyosis and pruritus	Spastic diplegia or quadriplegia	Intellectual disability	Ocular abnormalities	White matter encephalopathy	Preterm birth	Epilepsy	Reference
P1	F	2y	c.325 G > A (P) c.325 G > A (M)	p.Gly109Arg p.Gly109Arg	Homozygous	Yes	Yes	Yes	Yes	Yes	No	No	10
P2	M	1y	c.1157A > G (P) c.1294A > T (M)	p.Asn386Ser p.Arg432*	Compound heterozygous	Yes	Yes	Yes	Yes	Yes	Yes	No	10
P3	F	2y	c.1157A > G (P) IVS5-1del (M)	p.Asn386Ser	Compound heterozygous	Yes	Yes	Yes	/	Yes	No	Yes	11
P4	F	7y	c.1157A > G (P) c.1157A > G (M)	p.Asn386Ser p.Asn386Ser	Homozygous	Yes	Yes	Yes	/	Yes	No	No	11
P5	F	1y	c.1157A > G (P) c.1157A > G (M)	p.Asn386Ser p.Asn386Ser	Homozygous	Yes	Yes	Yes	/	/	Yes	No	11
P6	F	9y	c.723C > G (P) c.1157A > G (M)	p.Cys241Trp p.Asn386Ser	Compound heterozygous	Yes	Yes	Yes	No	Yes	Yes	No	12
P7	F	2y	c.1157A > G (P) c.1157A > G (M)	p.Asn386Ser p.Asn386Ser	Homozygous	Yes	Yes	Yes	Yes	Yes	Yes	No	13
P8	M	/	c.1157A > G (P) c.1157A > G (M)	p.Asn386Ser p.Asn386Ser	Homozygous	/	/	Yes	/	/	/	/	8
P9	F	5y	c.1157A > G (P) c.779delA (M)	p.Asn386Ser p.Lys260Argfs*6	Compound heterozygous	Yes	Yes	Yes	/	Yes	No	Yes	14
P10	F	4y	c.1157A > G c.207delT	p.Asn386Ser p.Phe70Leufs*37	Compound heterozygous	Yes	Yes	Yes	/	No	/	No	15
P11	M	2y	c.798delG (P) c.1241_1242del (M)	p.Glu267Asnfs*7 p.His414Glnfs*3	Compound heterozygous	Yes	Yes	Yes	/	Yes	Yes	Yes	16
P12	F	8 m	c.207delT c.1157A > G	p.Phe70Leufs*37 p.Asn386Ser	Compound heterozygous	Yes	Yes	Yes	/	/	Yes	No	16
P13	F	3y	c.1157A > G (P) c.1157A > G (M)	p.Asn386Ser p.Asn386Ser	Homozygous	Yes	Yes	Yes	/	No	No	Yes	17
P14	M	1y	c.325 G > A (P) c.1157A > G (M)	p.Gly109Arg p.Asn386Ser	Compound heterozygous	Yes	Yes	Yes	No	No	Yes	Yes	18
P15	F	2y	c.1157A > G (P) c.1157A > G (M)	p.Asn386Ser p.Asn386Ser	Homozygous	Yes	Yes	Yes	/	/	No	Yes	19
P16	F	1y	c.1157A > G c.1309A > T	p.Asn386Ser p.Lys437*	Compound heterozygous	Yes	No	Yes	Yes	/	/	No	19
P17	M	1y	loss1(EXON:1–10) (P) c.1157A > G (M)	p.Asn386Ser	Compound heterozygous	Yes	Yes	Yes	Yes	Yes	/	No	20
P18	M	1y	c.325 G > A (P) c.798+1(IVS5)delG (M)	p.Gly109Arg	Compound heterozygous	Yes	/	Yes	/	Yes	/	No	20
P19	F	10y	c.1157A > G (P) c.421dup (M)	p.Asn386Ser p.Glu141Glyfs*24	Compound heterozygous	Yes	Yes	Yes	Yes	Yes	No	No	21
P20	M	3y	c.1157A > G (P) c.1157A > G (M)	p.Asn386Ser p.Asn386Ser	Homozygous	Yes	Yes	Yes	/	No	Yes	No	This article
P21	F	2 m	c.779delA (P) c.1157A > G (M)	p.Lys260Argfs*6 p.Asn386Ser	Compound heterozygous	Yes	Yes	Yes	Yes	Yes	Yes	No	This article
P22	M	2y	c.313_327delinsTG (P) c.313_327delinsTG (M)	p.Val105Trpfs*14 p.Val105Trpfs*14	Homozygous	Yes	Yes	Yes	No	No	Yes	No	This article

M, male; F, female; m mouths; y, years; P, paternal; M, maternal

**Fig. 2:**
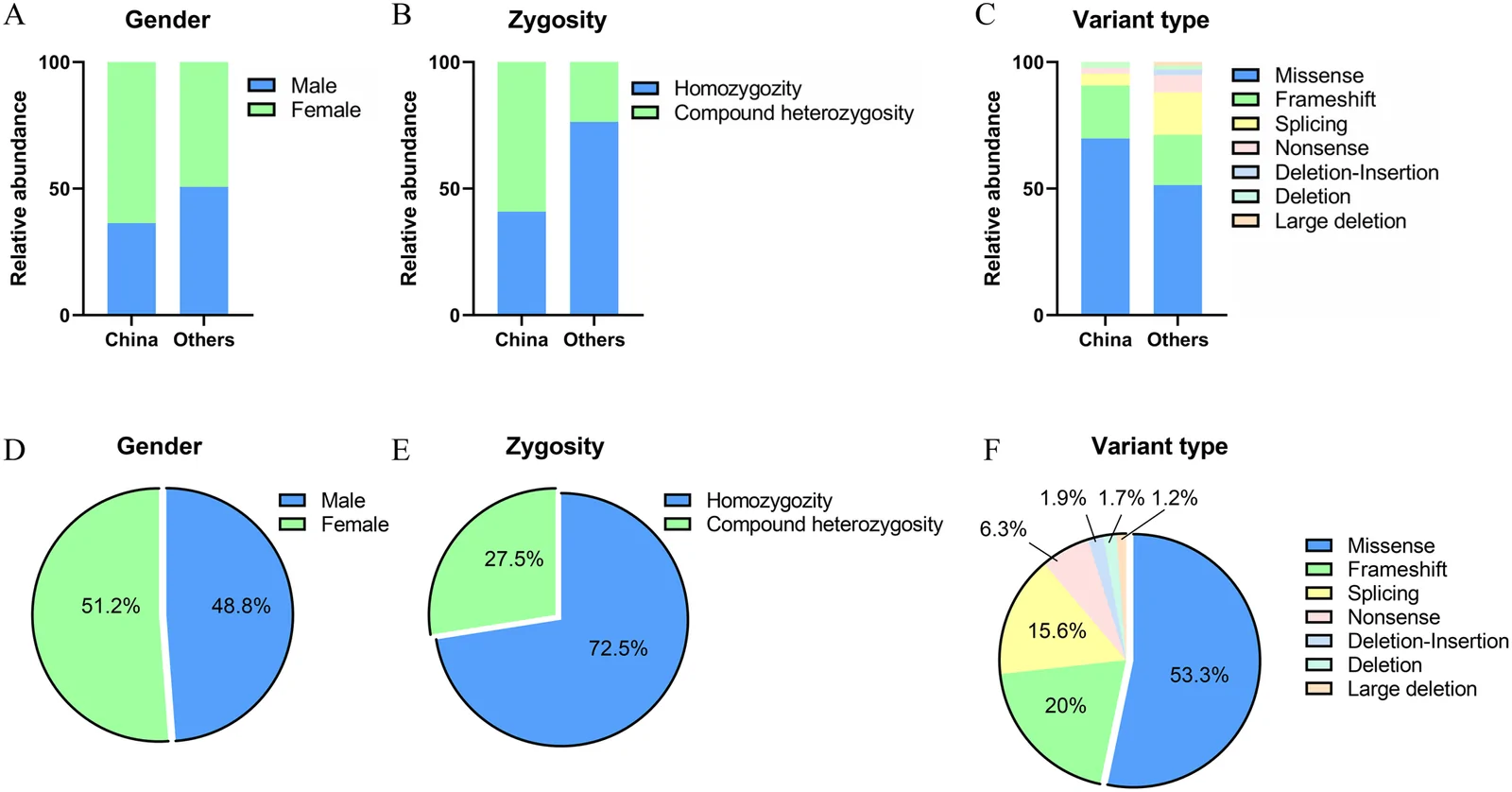
(**A**) Differences in gender composition between Chinese patients and patients from other countries (**B**) Differences in zygosity between Chinese patients and patients from other countries (**C**) Differences in types of gene mutations between Chinese patients and patients from other countries (**D**) The pooled analysis of gender ratio (**E**) The pooled analysis of zygosity ratio (**F**) The pooled analysis of proportion of mutation types

Among the 22 Chinese patients, 14 were female and 8 were male (63.6% vs 36.4%). Genetically, 13 were compound heterozygotes and 9 were homozygotes (59.1% vs 40.9%). There were 30 missense mutations (68.2%), 9 frameshift mutations (20.5%), 2 nonsense mutations (4.5%), 2 splice site mutations (4.5%), and 1 deletion mutation (2.3%). Compared with cohorts from other countries, Chinese patients showed a higher proportion of females, and compound heterozygosity was more common than homozygosity (Fig. 2A–C).

In the pooled analysis of all SLS patients, females accounted for 51.2% and males for 48.8% (Fig. 2D). Homozygous genotypes were more frequent than compound heterozygous genotypes (72.5% vs 27.5%; Fig. 2E). Regarding variant classes (synonymous mutations were excluded), missense variants were most common (53.3%), followed by frameshift variants (20.0%) and splice-site variants (15.6%) (Fig. 2F).

### Possible pathogenic mechanisms of the most common mutations in Chinese patients

**Fig. 3:**
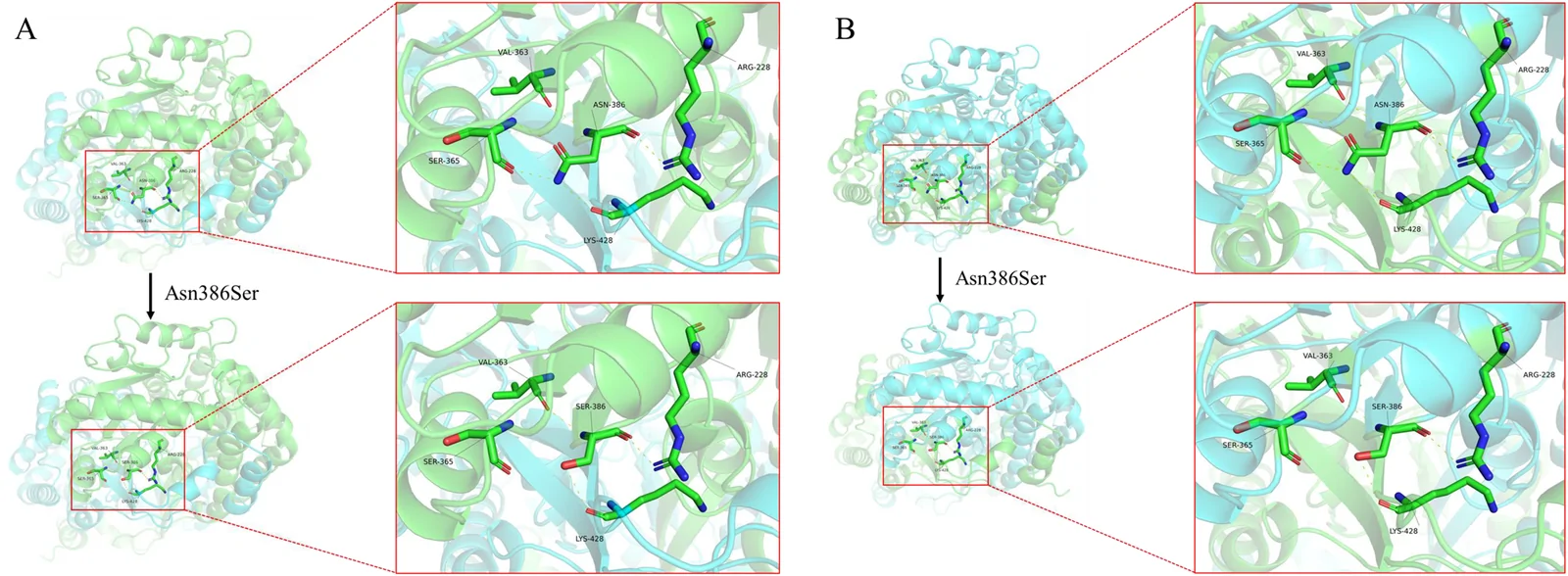
Three-dimensional structure of FALDH (wild type and mutant type), with two subunits shown in light green (**A**) and light blue (**B**) In the figure

Using published FALDH structures from the RCSB PDB (https://www.rcsb.org/) and PyMOL 3.3.6.1, we modeled local molecular interactions around affected residues. FALDH is a homodimer; in Fig. 3, the two subunits are shown in light green (Fig. 3A) and light blue (Fig. 3B). Figure 3 depicts the modeled 3D structure harboring the Asn386Ser (N386S) substitution. Carbon atoms are colored green, nitrogen blue, and oxygen red; hydrogen bonds are shown as yellow dashed lines. Substitution of Asn386 with Ser386 abolishes the hydrogen bond with Ser365, which is predicted to destabilize the local tertiary structure of the protein.

## Discussions

Globally, SLS is rare but has been reported across diverse geographic regions. Several recurrent mutations have been documented in patients from Europe [22], Brazil [23], and Egypt [24] with haplotype analyses suggesting founder effects and roles for consanguinity. For example, in the Brazilian population, the most common pathogenic variant is the c.1108-1 G > C splice-site mutation. The c.1157A > G missense variant—a prevalent allele in Japan—was also identified as the most frequent mutation in our Chinese SLS cohort. Overall, there is no substantial gender bias in SLS prevalence worldwide. Most reported cases carry homozygous variants, with a broad mutational spectrum in which missense changes predominate. In contrast, Chinese patients show some distinguishing features: a higher proportion of females and a greater frequency of compound heterozygous variants. Owing to the relatively small number of reported cases, there may be reporting bias in results such as sex distribution and genotype patterns.

SLS symptoms markedly impair quality of life and impose significant socioeconomic burden. At present, there is no curative therapy; management remains supportive and targeted toward symptom relief. Speech therapy and physiotherapy are important components of multidisciplinary care. Intrathecal baclofen injections have been reported to effectively lower muscle tone and alleviate spasticity [25]. Selected patients with severe joint contractures or intractable spasticity may benefit from orthopedic or neurosurgical interventions [26]. Dermatological symptoms, particularly ichthyosis, may respond to topical retinoids, but their use—especially at higher doses—is limited by a relatively high incidence of adverse effects [27]. Further research into pharmacologic approaches for SLS is urgently needed.

FALDH has a unique “gatekeeper” domain, consisting of a hydrophobic helix that covers the substrate entrance to the catalytic site channel [3]. Current work suggests that the hydrophobic helix/gatekeeper helix plays a crucial role in the function of FALDH and requires dimerization to position this helix at the substrate tunnel of adjacent subunits and to orient the two catalytic sites toward the lipid bilayer. These residues form two interaction pairs at the dimer interface. A mutation of either residue will lead to the loss of critical hydrogen bonds at the two symmetrical sites of the dimerization interface, resulting in significant instability of the dimer. N386S is a mutation located at the dimerization interface. After ASN-386 is replaced with SER-386, the hydrogen bond interacting with SER-365 is lost, which may lead to dimer instability and subsequent loss of function. Currently, FALDH catalyzes the oxidation of medium-chain fatty aldehydes to fatty acids, with activity against various saturated or unsaturated fatty aldehydes that are between 6 to 24 carbons in length [28]. Fatty aldehyde molecules that accumulate due to abnormal metabolism of ALDH3A2 are generally considered to react with important cellular components and cause cell damage [29]. Current research suggests that the oxidation of ether-derived fatty aldehyde [30], the leukotriene B4 (LTB4) metabolic pathway [31], phytol oxidation [32], and conversion of hexadecenal to hexadecenoic acid [33]could be the pathogenesis of SLS. These metabolic pathways may become drug targets for Sjögren-Larsson syndrome. Notably, preclinical research has shown that the aldehyde-trapping compound ADX-102 [2-(3-amino-6-chloro-quinolin-2-yl)-propan-2-ol] can prevent the formation of long-chain aldehyde adducts in cultured FALDH-deficient cells, protect cells from aldehyde-induced toxicity, and inhibit the reduction of fatty aldehydes to fatty alcohols. This suggests ADX-102 and similar compounds may represent promising avenues for pharmacological intervention in SLS [34]. Children with SLS are often born preterm. One of the metabolic pathways involved in SLS is the degradation of LTB4. FALDH deficiency can impair LTB4 degradation, resulting in elevated LTB4 levels. It has been reported that the preterm birth observed in SLS may be caused by increased secretion of LTB4 in fetal urine, which then enters the amniotic fluid [35]. We also agree with this hypothesis, as it provides new insight into the mechanism leading to preterm birth.

## Conclusion

Chinese SLS patients exhibit distinctive clinical and genetic patterns relative to other populations, with c.1157A > G being the most frequent variant. Structural modeling supports a potential conformational impact of these variants on FALDH.

## Electronic supplementary material

Below is the link to the electronic supplementary material.

Supplementary Material 1

## Data Availability

The datasets that underpin the conclusions of this study can be obtained from the corresponding author upon reasonable request
