# Supplementary material for: Clinical and genetic analysis of patients with Sjögren-Larsson syndrome in China
Source: Orphanet J Rare Dis. 2026 Jul 16;21:362. doi: 10.1186/s13023-026-04489-x (PMC13673612; doi:10.1186/s13023-026-04489-x)
Supplement: Supplementary file 1 [file 13023_2026_4489_MOESM1_ESM.pdf]

Supplementary Table. The genetic features of 178 SLS patients (excluding China) were retrieved from LOVD.

Indiviaual ID	Gender	Country	Allele(Parent1)		Allele（Parent2）		Zygosity
			DNA chang(cDNA)	Protein	DNA chang(cDNA)	Protein	
00064676	M	Japan	c.521del	p.Leu174Argfs*28	c.809del	p.Gly270Glufs*4	compound heterozygosity
00074600	F	Honduras	c.1309A>T	p.(Lys437*)	c.1309A>T	p.(Lys437*)	homozygozity
00079126	M	Honduras	c.1309A>T	p.(Lys437*)	c.1309A>T	p.(Lys437*)	homozygozity
00079361	F	France	c.471+1del	p.?	c.471+1del	p.?	homozygozity
00079569	F	France	c.472-1G>T	p.?	c.733G>A	p.(Asp245Asn)	compound heterozygosity
00079570	F	France	c.619dup	p.(Glu207Glyfs*11)	c.471+1del	p.?	compound heterozygosity
00079633	F	Algeria	c.370G>A	p.(Gly124Arg)	c.370G>A	p.(Gly124Arg)	homozygozity
00079634	M	Tunisia	c.648_649insAT	p.(Asp217Metfs*14)	c.648_649insAT	p.(Asp217Metfs*14)	homozygozity
00079874	M	Netherlands	c.798G>C	p.(Lys266Asn)	c.1297_1298del	p.(Glu433Argfs*3)	compound heterozygosity
00079916	M	Netherlands	c.25_50del	p.(Arg9Alafs*36)	c.943C>T	p.(Pro315Ser)	compound heterozygosity
00079917	M	Netherlands	c.943C>T	p.(Pro315Ser)	c.943C>T	p.(Pro315Ser)	homozygozity
00079918	F	Netherlands	c.80T>C	p.(Leu27Pro)	c.80T>C	p.(Leu27Pro)	homozygozity
00079919	F	Netherlands	c.798G>C	p.(lys266Asn)	c.1297_1298del	p.(Glu433Argfs*3)	compound heterozygosity
00079920	F	Netherlands	c.80T>C	p.(Leu27Pro)	c.1297_1298del	p.(Glu433Argfs*3)	compound heterozygosity
00080727	F	Turkey	c.487dup	p.(Ile163Asnfs*2)	c.487dup	p.(Ile163Asnfs*2)	homozygozity
00080754	M	Turkey	c.1384_1387del	p.(Glu462Asnfs*13)	c.1384_1387del	p.(Glu462Asnfs*13)	homozygozity
00080755	F	Turkey	c.1384_1387del	p.(Glu462Asnfs*13)	c.1384_1387del	p.(Glu462Asnfs*13)	homozygozity
00080756	F	Turkey	c.733G>A	p.(Asp245Asn)	c.733G>A	p.(Asp245Asn)	homozygozity
00080757	M	Netherlands	c.1297_1298del	p.(Glu433Argfs*3)	c.1297_1298del	p.(Glu433Argfs*3)	homozygozity
00080758	M	Netherlands	c.1297_1298del	p.(Glu433Argfs*3)	c.1297_1298del	p.(Glu433Argfs*3)	homozygozity
00081222	F	Netherlands	c.943C>T	p.(Pro315Ser)	c.1297_1298del	p.(Glu433Argfs*3)	compound heterozygosity
00081223	F	Netherlands	c.943C>T	p.(Pro315Ser)	c.1297_1298del	p.(Glu433Argfs*3)	compound heterozygosity
00081224	M	Netherlands	c.1297_1298del	p.(Glu433Argfs*3)	c.1297_1298del	p.(Glu433Argfs*3)	homozygozity
00081225	F	Netherlands	c.1297_1298del	p.(Glu433Argfs*3)	c.1297_1298del	p.(Glu433Argfs*3)	homozygozity
00081226	M	Netherlands	c.943C>T	p.(Pro315Ser)	c.551C>T	p.(Thr184Met)	compound heterozygosity
00081227	F	Netherlands	c.943C>T	p.(Pro315Ser)	c.551C>T	p.(Thr184Met)	compound heterozygosity
00081228	M	Spain	c.681-14T>C	p.(=)	c.681-14T>C	p.(=)	homozygozity
00081229	M	Turkey	c.835T>A	p.(Tyr279Asn)	c.835T>A	p.(Tyr279Asn)	homozygozity
00081230	M	Turkey	c.835T>A	p.(Tyr279Asn)	c.835T>A	p.(Tyr279Asn)	homozygozity
00081231	/	Turkey	c.1139G>A	p.(Ser380Asn)	c.1139G>A	p.(Ser380Asn)	homozygozity
00081232	F	Turkey	c.683G>A	p.(Arg228His)	c.683G>A	p.(Arg228His)	homozygozity
00081233	F	Japan	c.503_504dup	p.(Glu169Argfs*7)	c.1339A>G	p.(Lys447Glu)	compound heterozygosity
00081234	F	United States	c.-106175_*219565del	p.0?	c.-106175_*219565del	p.0?	homozygozity
00081235	F	United States	c.-835830_*581312del	p.0?	c.407C>T	p.(Pro136Leu)	compound heterozygosity
00081236	F	India	c.142G>T	p.(Asp48Tyr)	c.142G>T	p.(Asp48Tyr)	homozygozity
00081237	F	India	c.142G>T	p.(Asp48Tyr)	c.142G>T	p.(Asp48Tyr)	homozygozity
00081238	M	United States	c.798+1_798+5del	p.?	c.798+1_798+5del	p.?	homozygozity
00081239	F	United States	c.798+5_798+6del	p.?	c.798+5_798+6del	p.?	homozygozity
00081240	M	United States	c.798+5G>A	p.?	c.798+5G>A	p.?	homozygozity
00081241	F	United States	c.798+1del	p.?	c.798+1del	p.?	homozygozity
00081242	M	United States	c.1297_1298del	p.(Glu433Argfs*3)	c.1297_1298del	p.(Glu433Argfs*3)	homozygozity
00081243	M	United States	c.710G>A	p.(Cys237Tyr)	c.710G>A	p.(Cys237Tyr)	homozygozity
00081244	F	United States	c.1297_1298del	p.(Glu433Argfs*3)	c.1297_1298del	p.(Glu433Argfs*3)	homozygozity
00081245	F	United States	c.710G>A	p.(Cys237Tyr)	c.710G>A	p.(Cys237Tyr)	homozygozity
00081246	F	Sweden	c.943C>T	p.(Pro315Ser)	c.943C>T	p.(Pro315Ser)	homozygozity
00081247	M	Sweden	c.943C>T	p.(Pro315Ser)	c.943C>T	p.(Pro315Ser)	homozygozity
00081248	M	Sweden	c.943C>T	p.(Pro315Ser)	c.943C>T	p.(Pro315Ser)	homozygozity
00081249	M	Sweden	c.943C>T	p.(Pro315Ser)	c.943C>T	p.(Pro315Ser)	homozygozity
00081250	F	Sweden	c.943C>T	p.(Pro315Ser)	c.943C>T	p.(Pro315Ser)	homozygozity

00081251	F	Sweden	c.943C>T	p.(Pro315Ser)	c.943C>T	p.(Pro315Ser)	homozygozity
00081252	M	Sweden	c.943C>T	p.(Pro315Ser)	c.943C>T	p.(Pro315Ser)	homozygozity
00081253	F	Sweden	c.943C>T	p.(Pro315Ser)	c.943C>T	p.(Pro315Ser)	homozygozity
00081254	M	Sweden	c.943C>T	p.(Pro315Ser)	c.943C>T	p.(Pro315Ser)	homozygozity
00081255	F	Sweden	c.943C>T	p.(Pro315Ser)	c.943C>T	p.(Pro315Ser)	homozygozity
00081256	M	Sweden	c.943C>T	p.(Pro315Ser)	c.943C>T	p.(Pro315Ser)	homozygozity
00081257	M	Sweden	c.943C>T	p.(Pro315Ser)	c.943C>T	p.(Pro315Ser)	homozygozity
00081258	M	Sweden	c.943C>T	p.(Pro315Ser)	c.943C>T	p.(Pro315Ser)	homozygozity
00081259	M	Sweden	c.943C>T	p.(Pro315Ser)	c.943C>T	p.(Pro315Ser)	homozygozity
00081260	F	Sweden	c.943C>T	p.(Pro315Ser)	c.943C>T	p.(Pro315Ser)	homozygozity
00081261	F	Sweden	c.943C>T	p.(Pro315Ser)	c.943C>T	p.(Pro315Ser)	homozygozity
00081262	M	Sweden	c.943C>T	p.(Pro315Ser)	c.943C>T	p.(Pro315Ser)	homozygozity
00081263	M	Sweden	c.943C>T	p.(Pro315Ser)	c.943C>T	p.(Pro315Ser)	homozygozity
00081264	F	Sweden	c.943C>T	p.(Pro315Ser)	c.943C>T	p.(Pro315Ser)	homozygozity
00081266	F	Sweden	c.943C>T	p.(Pro315Ser)	c.943C>T	p.(Pro315Ser)	homozygozity
00081267	M	Sweden	c.943C>T	p.(Pro315Ser)	c.943C>T	p.(Pro315Ser)	homozygozity
00081268	M	Sweden	c.943C>T	p.(Pro315Ser)	c.943C>T	p.(Pro315Ser)	homozygozity
00081269	F	Sweden	c.943C>T	p.(Pro315Ser)	c.943C>T	p.(Pro315Ser)	homozygozity
00081270	F	Sweden	c.943C>T	p.(Pro315Ser)	c.943C>T	p.(Pro315Ser)	homozygozity
00081274	F	Italy	c.769dup	p.(Ile257Asnfs*6)	c.769dup	p.(Ile257Asnfs*6)	homozygozity
00081275	M	Italy	c.1094C>T	p.(Ser365Leu)	c.471+2T>G	p.?	compound heterozygosity
00081279	F	Japan	c.332G>A	p.(Trp111*)	636T>G	p.(Ser212Arg)	compound heterozygosity
00081280	F	Brazil	c.1108-1G>C	p.?	c.1108-1G>C	p.?	homozygozity
00081281	F	Brazil	c.1108-1G>C	p.?	c.1108-1G>C	p.?	homozygozity
00081282	M	Brazil	c.1108-1G>C	p.?	c.1108-1G>C	p.?	homozygozity
00081283	F	Brazil	c.1108-1G>C	p.?	c.1108-1G>C	p.?	homozygozity
00081284	F	Brazil	c.1108-1G>C	p.?	c.1108-1G>C	p.?	homozygozity
00081285	F	Brazil	c.1108-1G>C	p.?	c.1108-1G>C	p.?	homozygozity
00081286	F	Brazil	c.1108-1G>C	p.?	c.1108-1G>C	p.?	homozygozity
00081287	M	Brazil	c.1108-1G>C	p.?	c.1108-1G>C	p.?	homozygozity
00081288	M	Brazil	c.1108-1G>C	p.?	c.1108-1G>C	p.?	homozygozity
00081292	F	Israel	c.682C>T	p.(Arg228Cys)	c.682C>T	p.(Arg228Cys)	homozygozity
00081293	M	Israel	c.682C>T	p.(Arg228Cys)	c.682C>T	p.(Arg228Cys)	homozygozity
00081294	F	Israel	c.682C>T	p.(Arg228Cys)	c.682C>T	p.(Arg228Cys)	homozygozity
00081295	F	Israel	c.682C>T	p.(Arg228Cys)	c.682C>T	p.(Arg228Cys)	homozygozity
00081296	M	Israel	c.682C>T	p.(Arg228Cys)	c.682C>T	p.(Arg228Cys)	homozygozity
00081297	M	Israel	c.682C>T	p.(Arg228Cys)	c.682C>T	p.(Arg228Cys)	homozygozity
00081298	F	Japan	c.1087_1089del	p.(Val363del)	c.481del	p.(Ile161Leufs*14)	compound heterozygosity
00081299	M	Japan	c.1087_1089del	p.(Val363del)	c.481del	p.(Ile161Leufs*14)	compound heterozygosity
00081300	/	United States	c.286_296del	p.(Tyr96Thrfs*39)	c.1268G>A	p.(Arg423His)	compound heterozygosity
00081301	/	United States	c.1223del	p.(Gly408Glufs*20)	c.1223del	p.(Gly408Glufs*20)	homozygozity
00081302	/	United States	c.551C>G	p.(The184Arg)	c.551C>G	p.(The184Arg)	homozygozity
00081303	/	United States	c.942_944del	p.(Pro315del)	c.942_944del	p.(Pro315del)	homozygozity
00081304	/	United States	c.374_378del	p.(Ala125Glyfs*12)	c.529C>T	p.(Arg177*)	compound heterozygosity
00081305	/	United States	c.1108-1G>C	p.?	c.1108-1G>C	p.?	homozygozity
00081306	/	United States	c.1108-1G>C	p.?	c.1250_1265dup	p.(Arg423Tyrfs*19)	compound heterozygosity
00081307	/	United States	c.1223dup	p.(Ala409Serfs*7)	c.1223dup	p.(Ala409Serfs*7)	homozygozity
00081309	/	United States	c.1107+2T>G	p.?	c.1107+2T>G	p.?	homozygozity
00081310	/	United States	c.1139G>A	p.(Ser380Asn)	c.1139G>A	p.(Ser380Asn)	homozygozity
00081311	/	United States	c.299_365dup	p.(Ile123Alafs*38)	c.299_365dup	p.(Ile123Alafs*38)	homozygozity
00081313	M	United States	c.835T>A	p.(Tyr279Asn)	c.1297_1298del	p.(Glu433Argfs*3)	compound heterozygosity
00081315	F	Japan	c.1157A>G	p.(Asn386Ser)	c.1157A>G	p.(Asn386Ser)	homozygozity

00088870	/	Germany	c.733G>A<br>c.901G>C<br>c.906del	p.(Asp245Asn)<br>p.(Ala301Pro)<br>p.(Phe302Leufs*12)	c.1210_1443+2del<br>c.1446A>T	p.?<br>P.(=)	compound heterozygosity
00088943	/	Germany	c.1210_1443+2del<br>c.1446A>T	p.?<br>P.(=)	c.1210_1443+2del<br>c.1446A>T	p.?<br>P.(=)	homozygozity
00088944	/	Germany	c.1210_1443+2del<br>c.1446A>T	p.?<br>P.(=)	c.1210_1443+2del<br>c.1446A>T	p.?<br>P.(=)	homozygozity
00088945	/	Germany	c.471+1del	p.?	c.471+1G>C	p.?	compound heterozygosity
00088946	/	Germany	c.385+2T>C	p.?	c.1277T>G	p.(Leu426*)	compound heterozygosity
00088947	/	Germany	c.733G>A<br>c.901G>C<br>c.906del	p.(Asp245Asn)<br>p.(Ala301Pro)<br>p.(Phe302Leufs*12)	c.733G>A<br>c.901G>C<br>c.906del	p.(Asp245Asn)<br>p.(Ala301Pro)<br>p.(Phe302Leufs*12)	homozygozity
00088948	/	Germany	c.733G>A<br>c.901G>C<br>c.906del	p.(Asp245Asn)<br>p.(Ala301Pro)<br>p.(Phe302Leufs*12)	c.1297_1298del	p.(Glu433Argfs*3)	compound heterozygosity
00088949	/	Germany	c.835T>A	p.(Tyr279Asn)	c.1297_1298del	p.(Glu433Argfs*3)	compound heterozygosity
00088951	F	(United States)	c.710G>A	p.(Cys237Tyr)	c.710G>A	p.(Cys237Tyr)	homozygozity
00088954	M	Japan	c.1291_1292del	p.(Lys431Glus*5)	c.1157A>G	p.(Asn386Ser)	compound heterozygosity
00088955	F	Japan	c.1291_1292del	p.(Lys431Glus*5)	c.1157A>G	p.(Asn386Ser)	compound heterozygosity
00089013	M	(Spain)	c.28C>T	p.(Gln10*)	c.991_993del	p.(Glu331del)	compound heterozygosity
00089014	F	(Spain)	c.28C>T	p.(Gln10*)	c.991_993del	p.(Glu331del)	compound heterozygosity
00089024	M	(United States)	c.73C>T	p.(Gln25*)	c.551C>T	p.(Thr184Met)	compound heterozygosity
00089046	/	Sweden	c.103del	p.(Gln35Argfs*8)	c.943C>T	p.(Pro315Ser)	compound heterozygosity
00089047	/	Spain	c.317T>G	p.(Leu106Arg)	c.317T>G	p.(Leu106Arg)	homozygozity
00089048	/	Sweden	c.678C>G	p.(Cys226Trp)	c.678C>G	p.(Cys226Trp)	homozygozity
00089050	/	Sweden	c.864dup	p.(Lys289*)	c.943C>T	p.(Pro315Ser)	compound heterozygosity
00089052	/	Lebanon	c.941_943delinsGGGCTAAAAGTACTGTTGGGG	p.(Ala314_Pro315delinsGlyAlaLysSerThrValGlyAla)	c.941_943delinsGGGCTAAAAGTACTGTTGGGG	p.(Ala314_Pro315delinsGlyAlaLysSerThrValGlyAla)	homozygozity
00089054	/	Germany	c.1094C>T	p.(Ser365Leu)	c.1094C>T	p.(Ser365Leu)	homozygozity
00089056	/	Turkey	c.1234G>A	p.(Gly412Arg)	c.1234G>A	p.(Gly412Arg)	homozygozity
00089067	/	Switzerland	c.1297_1298del	p.(Glu433Argfs*3)	c.1297_1298del	p.(Glu433Argfs*3)	homozygozity
00089068	/	Germany	c.1297_1298del	p.(Glu433Argfs*3)	c.1297_1298del	p.(Glu433Argfs*3)	homozygozity
00094006	M	India	c.529C>T	p.(Arg177*)	c.126del	p.(Thr43Argfs*64)	compound heterozygosity
00094020	M	Egypt	c.641G>A	p.(Cys214Tyr)	c.641G>A	p.(Cys214Tyr)	homozygozity
00100433	F	United States	c.471+2T>G	p.?	c.1094C>T	p.(Ser365Leu)	compound heterozygosity
00100434	M	United States	c.798G>C	p.(Lys266Asn)	c.943C>T	p.(Pro315Ser)	compound heterozygosity
00100435	M	United States	c.386-6A>G	p.(=)	c.554G>C	p.(Gly185Ala)	compound heterozygosity
00108195	M	(Iran)	c.371_373del	p.(Gly124del)	c.371_373del	p.(Gly124del)	homozygozity
00110426	M	Spain	c.154_155del	p.(Ser52*)	c.536A>T	p.(Asp179Val)	compound heterozygosity
00110535	M	Israel	c.682C>T	p.(Arg228Cys)	c.682C>T	p.(Arg228Cys)	homozygozity
00111378	M	Israel	c.682C>T	p.(Arg228Cys)	c.682C>T	p.(Arg228Cys)	homozygozity
00113556	M	United States	c.682C>T	p.(Arg228Cys)	c.764del	p.(Asn255Ilefs*11)	compound heterozygosity
00113557	F	United States	c.710G>A	p.(Cys237Tyr)	c.710G>A	p.(Cys237Tyr)	homozygozity
00113558	F	Saudi Arabia	c.-58107_472-584del	p.(=)	c.-58107_472-584del	p.(=)	homozygozity
00144394	F	Iran	c.1242_1243del	p.(His414Glnfs*3)	c.1242_1243del	p.(His414Glnfs*3)	homozygozity
00144438	M	Iran	c.631A>G	p.(Lys211Glu)	c.631A>G	p.(Lys211Glu)	homozygozity
00144443	M	Iran	c.371_373del	p.(Gly124del)	c.371_373del	p.(Gly124del)	homozygozity
00144444	F	Iran	c.733G>A<br>c.901G>C<br>c.906del<br>c.909T>G	p.(Asp245Asn)<br>p.(Ala301Pro)<br>p.(Phe302Leufs*12)<br>p.(=)	c.733G>A<br>c.901G>C<br>c.906del<br>c.909T>G	p.(Asp245Asn)<br>p.(Ala301Pro)<br>p.(Phe302Leufs*12)<br>p.(=)	homozygozity
00144446	M	Iran	c.25_50del	p.(Arg9Alafs*36)	c.25_50del	p.(Arg9Alafs*36)	homozygozity
00144453	F	Iran	c.25_50del	p.(Arg9Alafs*36)	c.25_50del	p.(Arg9Alafs*36)	homozygozity
00144454	M	Iran	c.103C>T	p.(Gln35*)	c.103C>T	p.(Gln35*)	homozygozity
00144632	M	Korea	c.1157A>G	p.(Asn386Ser)	c.1157A>G	p.(Asn386Ser)	homozygozity
00144633	F	Greece	c.551C>T	p.(Thr184Met)	c.551C>T	p.(Thr184Met)	homozygozity

00164368	M	(Germany)	c.683G>A	p.(Arg228His)	c.683G>A	p.(Arg228His)	homozygozity
00164369	M	(Germany)	c.683G>A	p.(Arg228His)	c.683G>A	p.(Arg228His)	homozygozity
00164370	M	(Germany)	c.683G>A	p.(Arg228His)	c.683G>A	p.(Arg228His)	homozygozity
00164371	F	(Germany)	c.683G>A	p.(Arg228His)	c.683G>A	p.(Arg228His)	homozygozity
00164372	M	(Germany)	c.683G>A	p.(Arg228His)	c.683G>A	p.(Arg228His)	homozygozity
00164373	F	(Germany)	c.683G>A	p.(Arg228His)	c.683G>A	p.(Arg228His)	homozygozity
00164374	F	(Germany)	c.683G>A	p.(Arg228His)	c.683G>A	p.(Arg228His)	homozygozity
00164457	M	(Brazil)	c.1108-1G>C	p.?	c.798+5G>A	p.?	compound heterozygosity
00179418	M	Pakistan	c.10G>T	p.(Glu4*)	c.10G>T	p.(Glu4*)	homozygozity
00179419	M	Pakistan	c.10G>T	p.(Glu4*)	c.10G>T	p.(Glu4*)	homozygozity
00179420	M	Pakistan	c.10G>T	p.(Glu4*)	c.10G>T	p.(Glu4*)	homozygozity
00179454	M	Cyprus	c.941_943delinsGGGCTAAAAGTACTGTTGGGG	p.(Ala314_Pro315delinsGlyAlaLysSerThrValGlyAla)	c.941_943delinsGGGCTAAAAGTACTGTTGGGG	p.(Ala314_Pro315delinsGlyAlaLysSerThrValGlyAla)	homozygozity
00179456	M	Cyprus	c.941_943delinsGGGCTAAAAGTACTGTTGGGG	p.(Ala314_Pro315delinsGlyAlaLysSerThrValGlyAla)	c.941_943delinsGGGCTAAAAGTACTGTTGGGG	p.(Ala314_Pro315delinsGlyAlaLysSerThrValGlyAla)	homozygozity
00179458	F	Cyprus	c.941_943delinsGGGCTAAAAGTACTGTTGGGG	p.(Ala314_Pro315delinsGlyAlaLysSerThrValGlyAla)	c.941_943delinsGGGCTAAAAGTACTGTTGGGG	p.(Ala314_Pro315delinsGlyAlaLysSerThrValGlyAla)	homozygozity
00179510	F	(Portugal)	c.805del	p.(Tyr269Metfs*5)	c.805del	p.(Tyr269Metfs*5)	homozygozity
00179512	F	(Netherlands)	c.1297_1298del	p.(Glu433Argfs*3)	c.1297_1298del	p.(Glu433Argfs*3)	homozygozity
00179518	M	Japan	c.1202G>A	p.(Gly401Glu)	c.1202G>A	p.(Gly401Glu)	homozygozity
00180959	F	Japan	c.1157A>G	p.(Asn386Ser)	c.1157A>G	p.(Asn386Ser)	homozygozity
00180975	M	Turkey	c.1108-1G>C	p.?	c.1108-1G>C	p.?	homozygozity
00180976	M	Turkey	c.1108-1G>C	p.?	c.1108-1G>C	p.?	homozygozity
00180977	M	Turkey	c.24_25delinsTT	p.(Arg9*)	c.24_25delinsTT	p.(Arg9*)	homozygozity
00180978	F	Turkey	c.24_25delinsTT	p.(Arg9*)	c.24_25delinsTT	p.(Arg9*)	homozygozity
00180979	M	Turkey	c.1108-1G>C	p.?	c.1108-1G>C	p.?	homozygozity
00180980	F	Turkey	c.1108-1G>C	p.?	c.1108-1G>C	p.?	homozygozity
00180981	F	Morocco	c.325G>A	p.(Gly109Arg)	c.325G>A	p.(Gly109Arg)	homozygozity
00181090	F	(India)	c.953T>C	p.(Leu318Pro)	c.953T>C	p.(Leu318Pro)	homozygozity
00181091	M	(India)	c.680+1G>A	p.?	c.798+1del	p.?	compound heterozygosity
00181092	M	(India)	c.680+1G>A	p.?	c.798+1del	p.?	compound heterozygosity
00303631	M	Jordan	c.628G>A	p.(Gly210Arg)	c.628G>A	p.(Gly210Arg)	homozygozity
00303631	F	Jordan	c.628G>A	p.(Gly210Arg)	c.628G>A	p.(Gly210Arg)	homozygozity
00374184	/	India	c.125delG	p.(Thr43ArgfsTer64)	c.125delG	p.(Thr43ArgfsTer64)	homozygozity
00383873	F	Israel	c.682C>T	p.(Arg228Cys)	c.682C>T	p.(Arg228Cys)	homozygozity
00408100	F	United Arab Emirates	c.682C>T	p.(Arg228Cys)	c.682C>T	p.(Arg228Cys)	homozygozity
